# A novel ultrafast-low-dose computed tomography protocol allows concomitant coronary artery evaluation and lung cancer screening

**DOI:** 10.1186/s12872-018-0830-4

**Published:** 2018-05-08

**Authors:** Carlo Gaudio, Gennaro Petriello, Francesco Pelliccia, Alessandra Tanzilli, Alberto Bandiera, Gaetano Tanzilli, Francesco Barillà, Vincenzo Paravati, Massimo Pellegrini, Enrico Mangieri, Paolo Barillari

**Affiliations:** 1 Department of Cardiovascular Sciences, Sapienza University, Via del Policlinico 155, 00161 Rome, Italy; 2 Villa Mafalda Clinical Institute, Rome, Italy

**Keywords:** Computed tomography, Coronary artery disease, Lung cancer screening, Smoker, Ultrafast low-dose

## Abstract

**Background:**

Cardiac computed tomography (CT) is often performed in patients who are at high risk for lung cancer in whom screening is currently recommended. We tested diagnostic ability and radiation exposure of a novel ultra-low-dose CT protocol that allows concomitant coronary artery evaluation and lung screening.

**Methods:**

We studied 30 current or former heavy smoker subjects with suspected or known coronary artery disease who underwent CT assessment of both coronary arteries and thoracic area (Revolution CT, General Electric). A new ultrafast-low-dose single protocol was used for ECG-gated helical acquisition of the heart and the whole chest. A single IV iodine bolus (70–90 ml) was used. All patients with CT evidence of coronary stenosis underwent also invasive coronary angiography.

**Results:**

All the coronary segments were assessable in 28/30 (93%) patients. Only 8 coronary segments were not assessable in 2 patients due to motion artefacts (assessability: 98%; 477/485 segments). In the *assessable segments*, 20/21 significant stenoses (> 70% reduction of vessel diameter) were correctly diagnosed. Pulmonary nodules were detected in 5 patients, thus requiring to schedule follow-up surveillance CT thorax. Effective dose was 1.3 ± 0.9 mSv (range: 0.8–3.2 mSv). Noteworthy, no contrast or radiation dose increment was required with the new protocol as compared to conventional coronary CT protocol.

**Conclusions:**

The novel ultrafast-low-dose CT protocol allows lung cancer screening at time of coronary artery evaluation. The new approach might enhance the cost-effectiveness of coronary CT in heavy smokers with suspected or known coronary artery disease.

## Background

Cardiac computed tomography (CT) scan is an ideal diagnostic tool for identifying coronary artery disease in patients with low or intermediate risk [1]. In recent years, cardiac CT is being often performed in patients who are at high risk either for coronary artery disease or lung cancer. The update edition of the National Institute for Health and Care Excellence (NICE) guidelines recommends cardiac CT as the first-line diagnostic tool for patients with new-onset chest pain due to suspected CAD [2]. Also, symptomatic patients with known coronary artery disease and previous percutaneous coronary intervention who have an unclear stress test but whose presentation suggests a high likelihood of having an in-stent restenosis or a ‘de novo’ stenosis might benefit from cardiac CT [3]. In 2014, the U.S. Preventive Services Task Force recommended annual lung cancer screening with ultra-low dose computed tomography for current and former heavy smokers aged 55 to 80 years [4]. Indeed, lung cancer screening in patients with suspected or known coronary artery disease undergoing cardiac CT may provide the opportunity to implement recommendation for lung cancer screening in clinical practice [5].

The aim of this pilot study was to test the diagnostic ability and radiation exposure of a novel ultra-low-dose CT protocol that along with coronary artery evaluation allows lung screening with no increase in contrast or radiation dose. The new technique overcomes the limitation of a double dose of contrast and radiation usually needed to assess cardiac and lung regions during two different examinations.

## Methods

### Study population

We studied 30 current or former heavy smokers aged 55 to 79 years. All were symptomatic subjects with effort-induced or typical chest pain with suspected or known coronary artery disease. Subjects were excluded in case of contraindications to iodinated contrast such as allergies and chronic kidney failure, or if there was any suspicion of pregnancy (Table 1). All cases underwent cardiac CT for assessment of coronary arteries. Additionally, all individuals had CT scanning for early lung cancer detection. Invasive coronary angiography was performed subsequently in all patients who had evidence of ≥ 1 coronary stenosis (> 70% reduction of vessel diameter).

**Table 1:** Inclusion and exclusion criteria of the study patients

INCLUSION CRITERIA	
• Current or former heavy smoking habit	
• Age: 55–79 years	
• High risk of coronary artery disease	
• Previous diagnosis of coronary artery disease	
EXCLUSION CRITERIA	
• Age < 40 years	
• Diagnosis of acute coronary syndrome	
• History of allergic reactions	
• Irregular heart rate	
• Chronic renal failure (i.e., estimated glomerular filtration rate ≤ 60 ml/min/1.73 m^2^)	
• Microalbuminuria	
• Lack of consent	

The study conforms to the ethical guidelines of the 1975 Declaration of Helsinki and was approved by the Institutional Board Review Committee of our Institution (ID Number: 671/2017/D). All participants gave their written informed consent for the entire study, including radiation exposure. The STARD (Standards for Reporting of Diagnostic Accuracy Studies) guidelines for reporting studies of diagnostic accuracy were followed [6].

### Study procedures

**Fig. 1:**
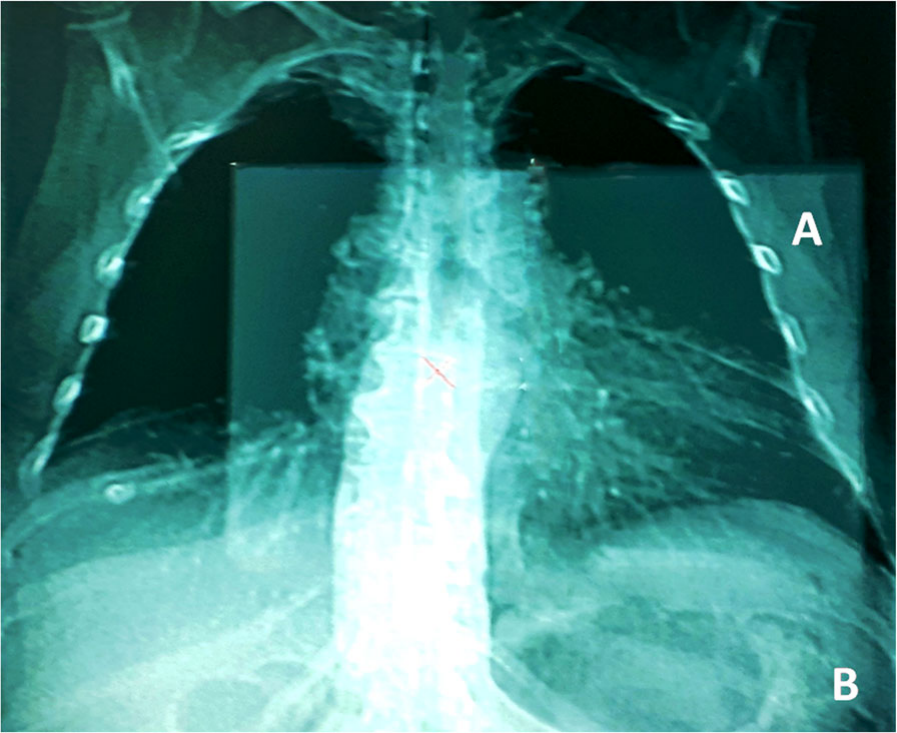
Ultrafast single protocol. The ECG-gated helical prospective acquisition started from the carena to the apex of the heart to evaluate coronary arteries (**a**, field of vew of cardiac scan), followed by fast, low dose acquisition, from pulmonary apex to the bases, on the whole chest (**b**, field of view of thoracic scan)

All subjects underwent simultaneous CT evaluation of coronary arteries and thoracic area (Revolution CT, General Electric, Boston, MA, US). The CT scanner operates in prospectively ECG-triggered sequential scanning mode, i.e. a tool adopted in spiral acquisitions in order to optimize radiation dose by adjusting the x-ray tube current. In case of heart rate > 70 beats/min, study subjects were given 50 mg of metoprolol orally 2 h before CT examination. ECG-gated helical prospective acquisition started from the carena to the apex of the heart to evaluate coronary arteries (100 kVp, variable mAs, thickness 0,625 mm, about 6 s apnea), followed by fast, low dose acquisition, from pulmonary apex to the bases, on the whole chest (100 to 120 kVp, auto mAs to adapt to the patient BMI, thickness 1,25 mm, 3 s apnea) (Fig. 1). A single IV iodine bolus (70–90 ml) was used. A bolus of 1 mL/kg of body weight (minimum of 70 mL) of iodixanol (Ultravist 370, Bayer HealthCare Pharmaceuticals, Berlin, Germany) followed by 80 mL of saline solution was continuously injected into a right antecubital vein through a catheter using a 5 mL/s flow rate. The segmental analysis of the coronary arteries was performed using the classification proposed by the American Heart Association which takes into consideration 16 segments [7]. When present, the intermediate branch (labeled as segment 17) was included in the analysis. Independently of reference vessel size, a coronary stenosis was considered significant if the diameter was ≥ 70%. The evaluation of any coronary stenosis was carried out, independently, by two investigators (FP and GP) who were blinded to the patients’ clinical characteristics. Coronary assessment was performed through a dedicated workstation (Vitrea2 FX, Vital Images, Plymouth, MN, USA) which allows the automatic identification of the coronary arterial borders [8]. When data analysis could not be performed in all coronary artery segments, the proportion of non-assessable segments was quantified.

Assessment of thoracic images obtained by CT scanning was performed by two investigators (AB and MP) with documented expertise in radiologic lung imaging. Pulmonary nodules were evaluated following the guidelines for screening of lung cancer published by the National Comprehensive Cancer Network (NCCN) [9]. A nodule was defined as a rounded or irregular opacity in the lung parenchyma, that was well or poorly defined, and had a diameter ≤ 3 cm. Also, pulmonary nodules were labeled as solid opacity, if there was a homogenous soft-tissue attenuation, or as ground-glass opacity, if there was a an area of hazy increased lung opacity with indistinct margins of pulmonary vessels. A positive test result in CT screening for lung cancer was defined by the finding of a noncalcified solid nodule ≥6 mm or a ground-glass nodule> 5 mm [9]. Contrast-to-noise ratio and signal-to-noise ratio were measured for quantitative assessment. Radiation doses delivered during CT scans were collected from patient CT acquisition protocols. Dose-length product (DLP) was recorded for each patient. Effective radiation dose (ED) was estimated using the formula “ED (mSv) ≈DLP x k”, where k is a conversion coefficient specific for adult chests (0.014 mSv/mGy × cm) [10].

### Quantitative coronary angiography

Invasive coronary angiography was accepted as the *reference* standard for the purpose of the study. In the week preceding CT scanning, all patients had left and right coronary angiography using the transfemoral or transradial approaches. In order to indentify coronary lesions with a significant (> 70%) stenosis, quantitative coronary angiography was used. Briefly, two investigators (FP and GT), blinded to the patients’ characteristics, performed all measurements independently. Coronary angiograms were evaluated off-line by means of a system that allows automated detection of the coronary artery edges (Cardiovascular Medical System, MEDIS Imaging Systems, Leiden, The Netherlands) [11]. Prior to coronary angiography, a bolus of intracoronary nitroglycerin (200 micrograms) was administered. Assessment of coronary wall morphology was done on angiographic views obtained after administration of nitroglycerin [12]. Of note, the investigators took into consideration all coronary lesions and irregularities that could be visually detected at coronary angiograms. When multiple coronary lesions were present in a single artery, they were labeled as distinct if separated by a normal tract of the arterial wall. The percent diameter stenosis was measured in the angiographic view that showed the most significant narrowing. For calibration, the catheter tip filled with contrast was used. This allowed to derive the reference diameter by interpolation. We measured all coronary segments that had a diameter > 2 mm showing a stenosis ranging between 20 and 100% [11]. Assessment of coronary stenosis was based on the formula: reference diameter-minimal lumen diameter/reference diameter × 100.

### Statistics

Data analysis included descriptive statistics. All data are reported as mean ± standard deviation, range, or percentage as appropriate. Statistical analysis was computed using SPSS 18.0.2 (IBM Corporation). The significance level for differences was set at *p* ≤ 0.05.

## Results

### Demographic

Thirty current or former heavy smoker subjects with chest pain and suspected CAD (20 men, mean age: 66 ± 9 years; range: 59–78 years) underwent simultaneous CT evaluation of the coronary arteries and the complete thoracic area (Table 2).

**Table 2:** Demographic, history, symptoms and indications to cardiac CT of the study population

No.	Heavy Smoking	Risk factors	Indication to cardiac CT	Cardiac CT findings	ICA Findings	Lung scan findings
1	Current	Hypertension, dyslipidemia	High-risk CAD	No stenosis	Not done	None
2	Former	Hypertension	High-risk CAD	LAD prox: 70% stenosis RCA: 90% stenosis	LAD prox: 80% stenosis RCA: 95% stenosis	Pulmonary nodule
3	Current	–	Positive EST	No stenosis	Not done	None
4	Former	Hypertension, dyslipidemia, diabetes	Previous PCI	RCA prox: 70% stenosis RCA mid: 90% stenosis	RCA prox: 80% stenosis RCA mid: 90% stenosis	None
5	Current	–	Suspected CAD	No stenosis	Not done	None
6	Former	Hypertension, dyslipidemia	High-risk CAD	LAD mid: 30% stenosis Cx mid: 90% stenosis	LAD mid: 80% stenosis Cx mid: 95% stenosis	Pulmonary nodule
7	Current	Diabetes	High-risk CAD	No stenosis	Not done	None
8	Current	Hypertension, dyslipidemia	Positive EST	LAD prox: 70% stenosis LAD distal: 70% stenosis	LAD prox: 90% stenosis LAD distal: 90% stenosis	None
9	Former	Diabetes, dyslipidemia	Previous PCI	Cx prox: 80% stenosis Cx mid: 90% stenosis	Cx prox: 70% stenosis Cx mid: 95% stenosis	None
10	Former	Hypertension	Suspected CAD	No stenosis	Not done	None
11	Current	Hypertension, dyslipidemia	High-risk CAD	No stenosis	Not done	None
12	Current	–	Positive EST	No stenosis	Not done	None
13	Former	Hypertension, dyslipidemia	Suspected CAD	No stenosis	Not done	Pulmonary nodule
14	Current	Hypertension, diabetes	Suspected CAD	No stenosis	Not done	None
15	Former	Hypertension, dyslipidemia	Positive EST	No stenosis	Not done	None
16	Former	Dyslipidemia	Suspected CAD	Cx prox: 80% stenosis Cx mid: 90% stenosis	Cx prox: 70% stenosis Cx mid: 95% stenosis	None
17	Current	Hypertension	Previous PCI	No stenosis	Not done	None
18	Current	Hypertension, dyslipidemia	Positive EST	No stenosis	Not done	None
19	Former	Hypertension	High-risk CAD	LAD prox: 70% stenosis LAD mid: 70% stenosis	LAD prox: 90% stenosis LAD mid: 90% stenosis	None
20	Former	Diabetes, dyslipidemia	Positive EST	No stenosis	Not done	Pulmonary nodule
21	Current	Hypertension, dyslipidemia	High-risk CAD	RCA prox: 80% stenosis RCA mid: 90% stenosis	RCA prox: 70% stenosis RCA mid: 95% stenosis	None
22	Current	Dyslipidemia	High-risk CAD	LAD prox: 70% stenosis OM prox: 90% stenosis RCA mid: 70% stenosis	LAD prox: 90% stenosis OM prox: 80% stenosis RCA mid: 90% stenosis	None
23	Former	Hypertension, dyslipidemia	High-risk CAD	No stenosis	Not done	None
24	Former	Dyslipidemia	Suspected CAD	No stenosis	Not done	None
25	Former	–	Positive EST	No stenosis	Not done	None
26	Current	Dyslipidemia	Positive EST	No stenosis	Not done	None
27	Current	Hypertension	Suspected CAD	No stenosis	Not done	None
28	Current	Hypertension, dyslipidemia	Suspected CAD	No stenosis	Not done	Pulmonary nodule
29	Former	Diabetes	High-risk CAD	No stenosis	Not done	None
30	Current	Hypertension, dyslipidemia	Previous PCI	RCA prox: In-stent 90% restenosis RCA mid: No in-stent restenosis RCA distal: No in-stent restenosis	RCA prox: In-stent 90% restenosis RCA mid: No in-stent restenosis RCA distal: No in-stent restenosis	None

*CAD Coronary artery disease, COPD Chronic obstructive pulmonary disease*, *CT* Computed tomography, *Cx* circumflex artery, *EST* Exercise stress test, *F* Female, *ICA* Invasive coronary angiography, *LAD* Left anterior descending, *M* male, *No*. Number, *PCI* Percutaneous coronary intervention, *RCA* Right coronary artery

### Coronary artery evaluation

**Fig. 2:**
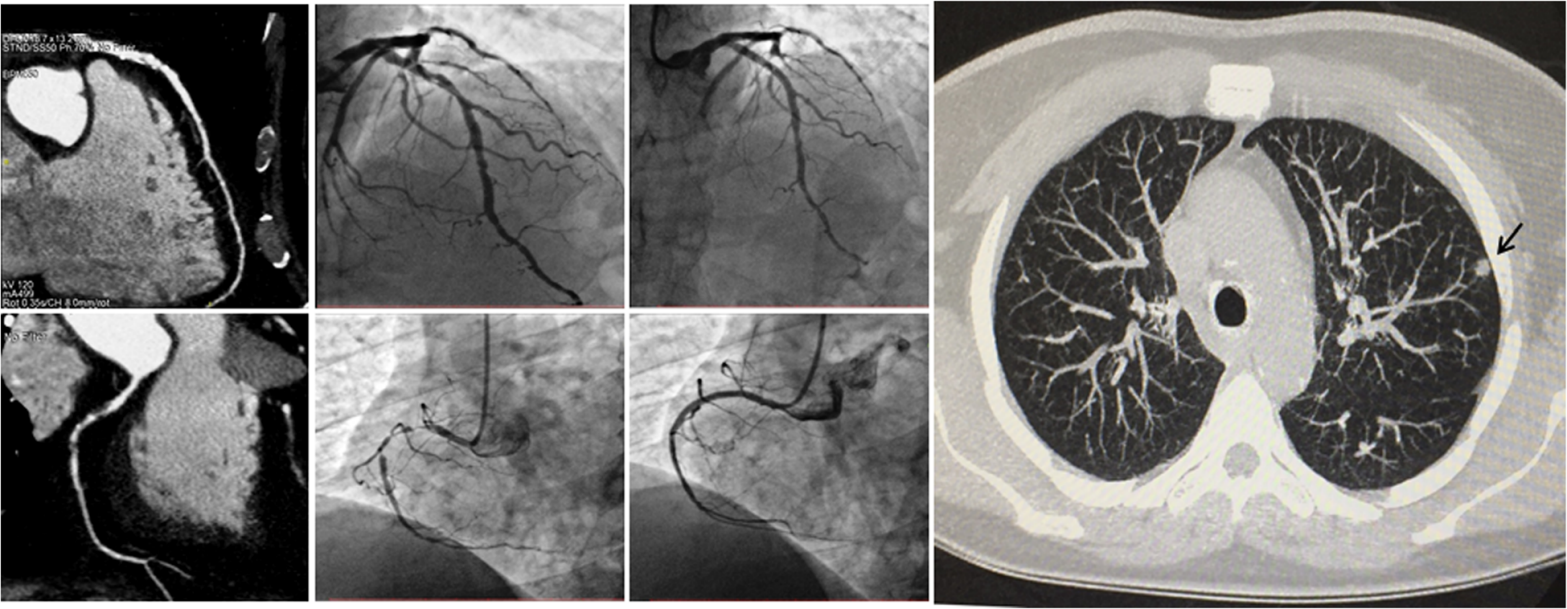
Representative case of coronary artery disease and lung cancer screening. The cardiac CT revealed a significant stenosis of the left anterior descending coronary artery (left upper panel), which was confirmed at coronary angiography (middle upper panel) and treated with percutaneous coronary intervention and stenting (right upper panel). Also, the right coronary artery showed a significant long stenosis in the proximal segment (left lower panel) that was confirmed at coronary angiography (middle lower panel) and treated with percutaneous coronary intervention and stenting (right lower panel). Ultra-low-dose CT images of the lungs showed a 6 mm pulmonary nodule in the left upper lobe (arrow, right panel)

**Fig. 3:**
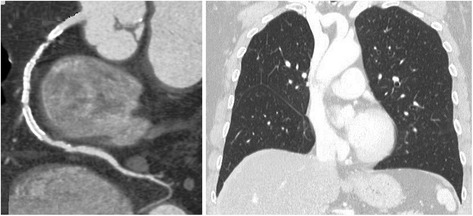
Representative case of follow-up evaluation of a patient with previous percutaneous coronary intervention and previous lung lobectomy. The cardiac CT revealed a significant in-stent restenosis in the proximal segment of the right coronary artery (left upper panel). Thoracic evaluation showed no recurrence 5 years after right upper lobectomy (right panel)

At CT scanning, coronary artery segments were judged to be assessable in 28/30 (93%) patients, as there were no step artifacts and motion artifacts were uncommon (3-point score: 0.59 ± 0.55) and did not affect coronary evaluation. In 2/30 (7%) patients, a total of 8 segments were judged to be non assessable because of motion artefacts. Accordingly, per-segment analysis disclosed an overall 98% assessability (477/485 segments). Coronary angiography was carried out in 10/30 (33%) patients who were found to have ≥ 1 coronary stenosis ≥ 70% at CT scanning (Fig. 2). Noteworthy, the invasive evaluation disclosed that CT scanning had correctly shown the majority (20/21) of significant (> 70%) coronary stenoses. In one patient only, coronary angiography found an 80% stenosis that was defined as non significant at CT scanning. Furthermore, cardiac CT showed significant in-stent restenosis in one of the patients who had had percutaneous coronary intervention (Fig. 3).

### Pulmonary CT evaluation

Pulmonary nodules were detected in 5 patients. All cases presented with solid nodules ≥ 6 mm (range: 6–11 mm), thus requiring to schedule follow-up surveillance CT thorax. Three other patients presented with solid nodules smaller than 6 mm, which were therefore considered negative according to National Comprehensive Cancer Network (NCCN) recommendations. No recurrence was found in a patient 5 years after right upper lobectomy (Fig. 2).

### Technical characteristics

The mean contrast-to-noise ratio and mean signal-to-noise ratio were respectively 12.5 ± 4.6 and 12.9 ± 3.3. Effective dose was 1.3 ± 0.9 mSv (range: 0.8–3.2 mSv). Noteworthy, no contrast or radiation dose increment was required with the new protocol as compared to conventional coronary CT protocol.

## Discussion

Cardiac CT offers a detailed anatomical assessment of CAD comparable to invasive coronary angiography [1]. Accordingly, CT coronary angiography has become rapidly an effective alternative to the traditional invasive angiography for screening and evaluating CAD. Indeed, the new generation of CT scanners has shown to yield high sensitivity and specificity in detecting angiographically *significant stenoses* [3]. Cardiac CT is said to be the diagnostic test to be preferred for evaluating patients with stable angina because of its favorable cost/benefit ratio. According to the guidelines of the National Institute for Health and Care Excellences (NICE), cardiac CT should be offered to all chest pain patients in whom CAD is suspected [2]. Especially in case of a high pre-testing cardiovascular risk profile, cardiac CT has been shown by randomized controlled trials to improve detection of CAD when incorporated in chest pain pathways [13, 14]. Of note, subjects with high cardiac risk are also current or former smokers and therefore have also a high-risk of lung cancer. Indeed, tobacco is a major risk factor for both CAD and lung cancer [15, 16], and previous studies have already ascertained that patients with coronary or cerebrovascular atherosclerosis are more likely to develop lung cancer [17].

Lung cancer remains the most common cancer in men and the third most common in women [18]. Early diagnosis is an important tool to reduce morbidity and mortality, and CT screening demonstrated a 20% decrease in the lung cancer mortality for high-risk populations such as heavy-smokers (> 30 pack/year) from 55 to 74 years [19, 20]. On the basis of available findings, the US Preventive Services Task Force now recommends annual lung cancer screening with ultra-low dose computed tomography for current and former heavy smokers aged 55 to 80 years.

With this background, there is an increased awareness that high-risk subjects undergoing imaging for cardiovascular conditions could also benefit from lung cancer screening. Radiation exposure has long been felt as a major limitation of CT screenings, but recent investigations have shown that ultra-low-dose CT is safer to screen high-risk patients [21–23].

Recently, it has been shown that associating a chest ultra-low-dose CT scan to the cardiac CT protocol for patients with suspected CAD is useful for lung cancer screening [5]. Our investigation confirms and extends this previous finding, as it shows that ultra-lo-dose CT is effective and safe for simultaneous CAD and lung cancer screening. Indeed, we report the first-in-man application of a novel ultra-low-dose CT protocol that allows simultaneous coronary artery and lung screening. The results obtained in the first series of 30 high-risk subjects that underwent the novel examination show that either coronary artery and lung evaluations were feasible. Noteworthy, no contrast or radiation dose increment was required as compared to conventional coronary CT protocol. The novel technique overcomes the limitation of a double dose of contrast and radiation usually needed to assess cardiac and lung regions during two different examinations.

Our study has some limitations. The major limitation lies on sample size. Even with important preliminary results, larger studies following up more patients for longer periods are needed to confirm the role of the novel ultrafast single protocol for lung cancer screening especially for reduction in mortality. Some studies have shown a reduced diagnostic performance to detect pulmonary nodules for obese patients undergoing fast-low-dose CT protocol, as higher body mass indexes are associated with increased image noise [23, 24]. A further limitation is constituted by the lack of a control group. As a consequence, we were unable to compare the novel ultra-low dose CT protocol with the standard CT scans.

## Conclusions

The new ultrafast-low-dose CT protocol seems to be effective and safe for simultaneous coronary artery evaluation and lung cancer screening. Such an approach may enhance the cost-effectiveness of coronary CT in heavy smokers with suspected CAD. Further studies are needed to assess the potential of the novel protocol to reduce cardiovascular and pulmonary morbidity and mortality in clinical practice.

## Abbreviations

CAD: Coronary artery disease
CT: Computed tomography
ECG: Electrocardiogram

## References

[1] Gaudio C, Pelliccia F, Evangelista A, Tanzilli G, Paravati V, Pannarale G (2013). 320-row computed tomography coronary angiography vs. conventional coronary angiography in patients with suspected coronary artery disease: a systematic review and meta-analysis. Int J Cardiol.

[2] Moss AJ, Williams MC, Newby DE, Nicol ED (2017). The updated NICE guidelines: cardiac CT as the first-line test for coronary artery disease. Curr Cardiovasc Imaging Rep.

[3] Janne d'Othee B, Siebert U, Cury R, Jadvar H, Dunn EJ, Hoffmann U (2008). A systematic review on diagnostic accuracy of CT-based detection of significant coronary artery disease. Eur J Radiol.

[4] Moyer VA (2014). U.S. Preventive Services Task Force. Screening for lung cancer: US Preventive Services Task Force Recommendation statement. Ann Intern Med.

[5] Zanon M, Pacini GS, de Souza VVS, Marchiori E, Meirelles GSP, Szarf G (2017). Early detection of lung cancer using ultra-low-dose computed tomography in coronary CT angiography scans among patients with suspected coronary heart disease. Lung Cancer.

[6] Bossuyt PM, Reitsma JB, Bruns DE, Gatsonis CA, Glasziou PP, Irwig LM (2003). Standards for reporting of diagnostic accuracy. The STARD statement for reporting studies of diagnostic accuracy: explanation and elaboration. Ann Intern Med.

[7] Austen WG, Edwards JE, Frye RL, Gensini GG, Gott VL, Griffith LS (1975). A reporting system on patients evaluated for coronary artery disease: report of the ad hoc Committee for Grading of coronary artery disease, council on cardiovascular surgery. American Heart Association. Circulation.

[8] Dewey M, Schnapauff D, Laule M, Lembcke A, Borges AC, Rutsch W (2004). Multislice CT coronary angiography: evaluation of an automatic vessel detection tool. Rofo.

[9] American Association of Physicists in Medicine. The Measurement, Reporting and Management of Radiation Dose in CT. Report 96, AAPM Task Group 23 of the Diagnostic Imaging Council CT Committee. 2008. https://www.aapm.org/pubs/reports/RPT_96.pdf. Accessed 5 May 2018.

[10] Lindell RM, Hartman TE, Swensen SJ, Jett JR, Midthun DE, Tazelaar HD (2007). Five-year lung cancer screening experience: CT appearance, growth rate, location, and histologic features of 61 lung cancers. Radiology.

[11] Waters D, Lespe’rance J, Craven TE, Hudon G, Gillam LD (1993). Advantages and limitations of serial coronary arteriography for the assessment of progression and regression of coronary atherosclerosis: implications for clinical trials. Circulation.

[12] Reiber JH, Serruys PW, Kooijman CJ, Wijns W, Slager CJ, Gerbrands JJ (1985). Assessment of short-, medium-, and long-term variations in arterial dimensions from computer-assisted quantitation of coronary cineangiograms. Circulation.

[13] Douglas PS, Hoffmann U, Patel MR, Mark DB, Al-Khalidi HR, Cavanaugh B (2015). Outcomes of anatomical versus functional testing for coronary artery disease. N Engl J Med.

[14] SCOT-HEART investigators (2015). CT coronary angiography in patients with suspected angina due to coronary artery disease (SCOTHEART): an open-label, parallel group multicentre trial. Lancet.

[15] Ambrose JA, Barua RS (2004). The pathophysiology of cigarette smoking and cardiovascular disease: an update. J Am Coll Cardiol.

[16] Jee SH, Suh I, Kim IS, Appel LJ (1999). Smoking and atherosclerotic cardiovascular disease in men with low levels of serum cholesterol: the Korea medical insurance corporation study. JAMA.

[17] Whitlock MC, Yeboah J, Burke GL, Chen H, Klepin HD, Hundley WG. Cancer and its association with the development of coronary artery calcification: an assessment from the multi-ethnic study of atherosclerosis. J Am Heart Assoc. 2015;4(11):e002533. 10.1161/JAHA.115.002533 PMC4845242 26553214

[18] Dela Cruz CS, Tanoue LT, Matthay RA (2011). Lung cancer: epidemiology, etiology, and prevention. Clin Chest Med.

[19] Cole P, Morrison AS (1980). Basic issues in population screening for cancer. J Natl Cancer Inst.

[20] Patz EF, Goodman PC, Bepler G (2000). Screening for lung cancer. N Engl J Med.

[21] Ebner L, Bütikofer Y, Ott D, Huber A, Landau J, Roos JE (2015). Lung nodule detection by microdose CT versus chest radiography (standard and dual-energy subtracted). AJR Am J Roentgenol.

[22] Huber A, Landau J, Ebner L, Bütikofer Y, Leidolt L, Brela B (2016). Performance of ultralow-dose CT with iterative reconstruction in lung cancer screening: limiting radiation exposure to the equivalent of conventional chest X-ray imaging. Eur Radiol.

[23] Kim Y, Kim YK, Lee BE, Lee SJ, Ryu YJ, Lee JH (2015). Ultra-low-dose CT of the thorax using iterative reconstruction: evaluation of image quality and radiation dose reduction. AJR Am J Roentgenol.

[24] Lee JY, Chung MJ, Yi CA, Lee KS (2008). Ultra-low-dose MDCT of the chest: influence on automated lung nodule detection. Korean J Radiol.

